# Special Issue on Cancer Smart Nanomedicine

**DOI:** 10.3390/cancers15225344

**Published:** 2023-11-09

**Authors:** Marina Pinheiro, Ammad Ahmad Farooqi

**Affiliations:** 1 LAQV, REQUIMTE, Department of Chemistry, Faculdade de Farmácia, Universidade do Porto, Rua de Jorge Viterbo Ferreira, 228, 4050-313 Porto, Portugal; 2 Life and Health Sciences Research Institute (ICVS), School of Medicine, University of Minho, 4710-057 Braga, Portugal; 3 Institute of Biomedical and Genetic Engineering (IBGE), Islamabad 54000, Pakistan

In this Special Issue entitled “*Cancer Smart Nanomedicine*”, we have gathered high-quality contributions related to the fascinating field of nanomedicine. The Special Issue contains eight articles, namely four research articles and four reviews. The complete description of each study and the main results are presented in the full manuscripts and readers are cordially invited to explore the contents in detail.

In the manuscript authored by Klebowski et al., the authors provide scientific proof related to bimetallic palladium–platinum nanoparticles with different nanostructures to improve the effectiveness of therapy, while reducing the necessary total dose of the radiation used. In this study, the authors provide evidence about the design and development of nanomaterials to modulate their radiosensitizing potential and enhance the effectiveness of cancer therapeutics [1].

In the manuscript written by Jugel et al., the authors detail how they developed cyclodextrein-modified poly(propylene imine) nanocarriers modified to specifically target minicircle DNA, targeting tumor cells. The authors found long-term expression of a therapeutic p53 gene in tumor cells [2].

The work of Katifelis et al. tested whether the developed nanoparticles, composed of gold and silver, could induce programmed cell death over other pathways, despite apoptosis. The authors demonstrated that necroptosis and pyroptosis occurred, and therefore the nanoparticles induced promising anticancer effects. These exciting aspects warrant further research [3].

The manuscript of Lafuente-Gómez details the development of a smart gemcitabine delivery system based on magnetic nanoparticles for the treatment of pancreatic cancer. The developed formulation proved to prevent the unspecific binding of proteins, reduced the cytotoxic effect of the drug in non-cancerous cells, and improved internalization in pancreatic cancer cells, and its activity was synergistically enhanced in combination with magnetic hyperthermia [4].

The review of Wahnou et al. highlights the potential use of natural compounds with anticancer properties as potential therapeutic agents for the treatment of various cancers, including colorectal cancer. This manuscript provides a comprehensive review about the potential use of nanoparticles to overcome the pharmacokinetic obstacles of polyphenols as anticancer drugs [5].

The review written by Dissanayake et al. provides an overview of the recent progress toward the use of nanotherapeutics for the treatment of metastatic breast cancer. In this review, the authors discuss in detail how nanotechnology can be used in combination with existing treatments [6].

The review written by Chuang et al. describes the functions of exosomal long non-coding RNAs, sponging, and exosomal processing in anticancer processes. This review also discusses future directions regarding the application of natural products in the regulation of non-coding RNA-loaded exosomes [7].

The review written by Ghidini et al. highlights the most recent developments in the field of nanomedicine RNA-associated therapies for the treatment of cancer. The ongoing clinical trials using RNA-loaded nanoparticles for cancer therapy are also described, with some promising formulations in phase I and II [8].
